# Prevalence and factors associated with hypertension among rural community dwellers in a local government area, South West Nigeria

**DOI:** 10.4314/ahs.v21i1.12

**Published:** 2021-03

**Authors:** Owigho P Opreh, Temitope O Olajubu, Kunmi J Akarakiri, Vojtech Ligenza, John T Amos, Adebanke V Adeyeye, Olufunke Z Oyelade, Funmilayo C Oyewole

**Affiliations:** Department of Family Medicine, Seventh Day Adventist Hospital, Lagere, Ile-Ife

**Keywords:** Prevalence, hypertension, rural, community, Nigeria

## Abstract

**Background:**

Many African countries including Nigeria are said to be at various stages of an epidemiological transition from communicable to non-communicable diseases (NCD).

**Objective:**

This study determined the current pattern and correlates of hypertension among adults in some rural communities in South West Nigeria.

**Methods:**

It was a descriptive cross-sectional study of 1012 individuals across 16 rural communities. The respondents' blood pressure, weight, height and waist circumference were measured. Bivariate and multivariate analyses were done.

**Results:**

Among the participants, 461 (45.6%) had hypertension out of whom 217 (47.1%) and 244 (52.9%) had stage 1 and stage 2 hypertension respectively. The systolic, diastolic and mean arterial blood pressures increased with age. The significant predictors of hypertension were; increasing age (p<0.001), higher waist circumference (p = 0.01) and overweight / obesity (p = 0.03). While systolic blood pressure (SBP) had the strongest correlation with age, waist circumference (WC) was the strongest correlate of diastolic blood pressure (DBP).

**Conclusion:**

Despite being a rural population, there was a high prevalence of hypertension in the study area.

## Introduction

Hypertension is the world's leading risk factor for global disease burden and it is projected to affect approximately 1.6 billion adults worldwide by 2025^1^–^3^. It is a very significant modifiable risk factors for cardiovascular and renal diseases^1^. Existing data suggest that hypertension has increased in economically developing countries in recent years while it remained stable in many developed countries^2^,^4^.

Most sub-Sahara African countries including Nigeria are said to be at various stages of an epidemiological transition from communicable to non-communicable diseases (NCD)^5^–^8^. However, as highlighted in a systematic review by Akinlua et al., many of the existing studies have largely focused on the urban population^8^. The rural communities tend to be underserved and under-penetrated in terms of health care services^9^. This contributes to the unmet needs for primary and secondary prevention of NCDs in the rural areas.

Hypertension does not usually present with specific symptoms until complications begin to set in, hence its identification is usually through screening^5^. It is important that epidemiologic data are regularly updated in view of the fact that such data are crucial for the continuous design and implementation of effective strategies for the prevention and control of hypertension among the different segments of the population. There is therefore the need for continuous generation of relevant empiric evidence aimed at apprpriate policy response.

This study thus determined the pattern and correlates of high blood pressure among adults living in the rural communities of a local government area in Osun State, South West Nigeria. This will hopefully provide additional useful information to policy makers and relevant stakeholders. It will also contribute to the continuously updated database about the disease nationally and globally.

## Methods

### Study setting and subjects

This descriptive cross-sectional study was carried out in Ife East local government area (LGA) of Osun state, South-Western Nigeria. Ife East was purposively selected out of the 30 LGAs in the state. The 2016 estimated population based on the 2006 census and a national annual population growth rate of 2.5% is 259,700^10^.

The LGA is divided into 10 administrative wards, out of which three wards were randomly selected for the study. Participants were recruited from all the major rural communities in the selected wards totaling 16 communities. Compared to general population, the population of the rural communities were tilted towards a predominance of the middle aged and elderly due to rural-urban migration by many of the younger ones.

Each of the communities were estimated to have about 100 adults aged 20 years and above. It was a consecutive house to house survey. All eligible and consenting adults in every household were recruited until an average of 60 respondents were enrolled in each community. Pregnant women were excluded.

The calculated minimum sample size, at 95% confidence level and 5% margin of error, was 378 using a previous prevalence of 46.4% from a similar study^5^. We however aimed at recruiting at least 1000 participants to enhance the precision of the study. Eventually, a total of 1012 consenting adults aged 20 years and above were included in the study. Ethical approval was obtained from the Ethics Committee of the Seventh Day Adventist Hospital, Ile-Ife and informed consent was given by all participants. In each community, the permission of the traditional ruler or the key leader was obtained.

### Data collection

Data collection was done in line with the World health Organization's STEP-wise approach^11^. This was done by trained nurses who were supervised by a medical doctor. The team had training sessions and a guideline for data collection was prepared. The guideline was used as reference on the field. The proper calibration of the sphygmomanometers used for the study was ensured.

### Measurement of blood pressure

Each respondent's blood pressure was measured with Accosson® Mercury sphygmomanometer within the same time frame each day. The blood pressure reading was taken in sitting position after the respondent has rested for about ten minutes. The participants were informed, and it was ascertained that coffee, alcohol or cigarette was not used, nor was any physical exercise done least 30 minutes before the measurement. An appropriate sized cuff was used and the bell of the stethoscope was placed about 2cm above the antecubital fossa. The cuff was inflated to 20mmHG above the palpated systolic blood pressure and thereafter deflated gradually. The first sound (Korotkoff phase 1) and the disappearance of the sound (phase 5) were taken as the systolic blood pressure (SBP) and diastolic blood pressure (DBP) respectively. Two readings were taken at an interval of about five minutes and the average was recorded. The supervisor oversaw the measurements.

A respondent was defined as having hypertension if he/she had a systolic blood pressure of ≥ 140mmHg and/or diastolic blood pressure reading of ≥ 90mmHg or was on pharmacological treatment for hypertension within the preceding two weeks^5^,^7^,^12^. Isolated Systolic hypertension (ISH) was said to be present when only the SBP was elevated and Isolated Diastolic Hypertension (IDH) was diagnosed if only the DBP was elevated. Furthermore, with regards to the severity or stage of hypertension, participants were categorized thus: normal: SBP < 120mmHg and DBP < 80mmHg, pre-hypertension: SBP of 120–139mmHg and/or DBP of 80–89, stage 1 hypertension: SBP of 140–159mmHg and/or DBP of 90–99mmHg, stage 2 hypertension: SBP ≥ 160mmHg and/or DBP ≥ 100mmHg^12^.

The weight, height and waist circumference were also measured. The weight was measured in light clothing to the nearest 0.1 kilograms without any accessories such as shoes, purses, cell phones, etc. The weighing scale was regularly adjusted to reset it to zero. The height was measured without foot-wears to the nearest 0.5 centimeter using a ruler attached to the wall. The body mass index (BMI) – defined as the weight in kilogram divided by the square of the height in meters – was calculated. Based on the BMI, the respondents were classified as either obese (BMI ≥ 30kg/m^2^), overweight (BMI 25 -29.9 kg/m^2^) or not (BMI <25kg/m^2^). The waist circumference (WC) was measured at the midpoint between the sub-costal margin and the iliac crest. Abdominal obesity was defined as waist circumference of ≥ 102 cm for men and ≥ 88cm in women^13^.

### Data analysis

Data was analyzed using the IBM SPSS (version 22). Descriptive statistics was used to summarize the data. At the bivariate level, the Pearson chi-square test was used to determine the significance of association between hypertension and some categorical variables. The differences in some continuous variables based on gender were assessed by Students' t-test while Pearson's correlation was done to determine the degree of relationship between quantitative variables. The factors that were significant at the bivariate level were included in a multivariate binary logistic regression model to determine those which independently predicted the presence of hypertension. Collinearity diagnostic tests were done and there was no multicollinearity among the variables that were included in the model. The results were expressed as odds ratio (OR). The level of significance for all the statistical analyses was set at p < 0.05.

## Results

All the eligible individuals who were approached for the study gave their consent to participate, yielding a 100% response rate. A total of 1012 individuals made up of 428 (42.3%) males and 584 (57.7%) females participated in the study. Their ages ranged from 20 to 103 years with a mean of 47.7 ± 16.7 years.

### Pattern of Blood pressure and Anthropometric measures

**Figure 1:**
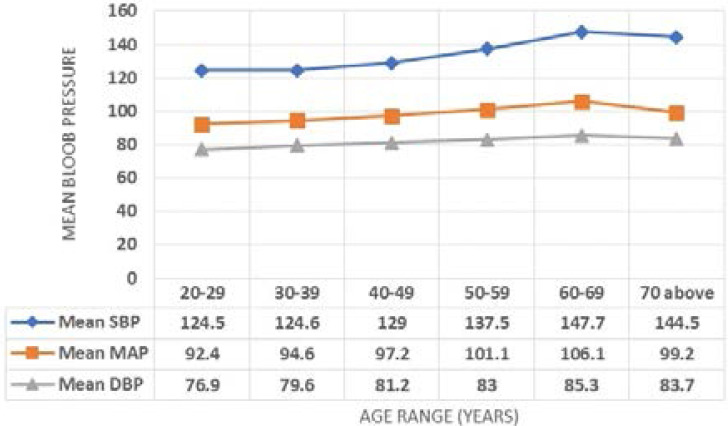
The pattern of Mean SBP, MAP and DBP across age groups

Among the participants, a total of 461 (45.6%) were defined as having hypertension by blood pressure measurement. Thirty four respondents (3.4%) reported the use of anti-hypertensive drugs while 427 (42.2%) were newly diagnosed or not on medication. However, all those who were on medication also had a SBP ≥ 140mmHg and/or DBP ≥ 90 mmHg, hence were part of the total of 461 (45.6%). Blood pressure was optimal in 183 (18.2%) respondents while pre-hypertension stage was seen in 367 (38.3%). The pattern of blood pressure across the different age groups is shown in figure 1. There was an increase in each of SBP, DBP and mean arterial pressure (MAP) with age before dropping slightly in those aged 70 years and above.

Analysis of the pattern of elevated blood pressure among the hypertensive subjects showed that 59.7% (275) of them had Systolic-Diastolic Hypertension (SDH) i.e. elevation of both the SBP and DBP, while 115 (24.9%) had isolated systolic hypertension (ISH), the remaining 15.2% had isolated diastolic hypertension (IDH). Furthermore, respondents who were 60 years and above had the highest (49.6%) prevalence of ISH. A comparison of gender distribution of some clinical parameters among the hypertensive respondents is shown on Table 1. When compared with women, men had significantly higher mean age, weight and height. The women however had significantly higher waist circumference and BMI. There were no significant differences in SBP, DBP and MAP between men and women.

**Table 1:** Clinical variables among respondents by gender

Variables	Men (n=428) Mean ± SD	Women (n=584) Mean ± SD	*P* value
Age (years)	49.0 ± 18.5	46.7 ± 15.1	0.035
Weight (kg)	59.3 ± 10.0	56.7 ± 11.3	< 0.001
Height (m)	1.66 ± 0.07	1.57 ± 0.06	< 0.001
Waist circumference (cm)	80.9 ± 9.0	82.3 ± 10.3	0.021
BMI (kg/m^2^)	21.6 ± 3.4	23.0 ± 4.3	< 0.001
SBP (mmHg)	134.7 ± 23.9	133.9 ± 25.5	0.596
DBP (mmHg)	82.5 ± 13.5	81.0 ± 13.3	0.086
Mean Arterial BP (mmHg)	99.9 ± 15.3	98.6 ± 15.7	0.204

Notes: SD – Standard deviation, BMI – Body Mass Index, SBP – Systolic Blood Pressure, DBP – Diastolic Blood Pressure

### Factors associated with hypertension

Among the respondents, hypertension was significantly associated with age (p<0.001), marital status (p<0.001), waist circumference (p<0.001) and BMI (p<0.001). [Table 2]. The prevalence of hypertension increased steadily from 11.8% in those aged 20–29 years, 39.7% in the 40–49 age group to the highest of 73.6% among those aged 60–69 years. The prevalence was 47% in men and 44.5% in women but this difference was not statistically significant (p = 0.44).

**Table 2:** Association between Hypertension and Socio-demographic variables

	Has hypertension n (%)	No hypertension n (%)	χ^2^	p value
**Age group**				
20 – 29	17 (11.8)	127 (88.2)	182.55	<0.001
30 – 39	48 (26.2)	135 (73.8)		
40 – 49	75 (39.7)	114 (60.3)		
50 – 59	109 (55.3)	88 (44.7)		
60 – 69	128 (73.6)	46 (26.4)		
70 and above	84 (67.2)	41 (32.8)		
**Gender**				
Male	201 (47.0)	227 (53.0)	0.60	0.44
Female	260 (44.5)	324 (55.5)		
**Marital status**				
Single	16 (21.1)	60 (78.9)	28.15	< 0.001
Married	385 (45.9)	453 (54.1)		
Widowed	60 (61.2)	38 (38.8)		
**WC**				
Normal	324 (40.4)	478 (59.6)	41.40	<0.001
High	137 (65.2)	73 (34.8)		
**BMI**				
< 25 kg/m^2^	334 (41.8)	466 (58.2)	25.79	< 0.001
Overweight	90 (56.2)	70 (43.8)		
Obese	37 (71.2)	15 (28.8)		

As shown in table 3, all the variables assessed had significant correlation with both SBP and DBP. While SBP had the strongest correlation with age, waist circumference was the strongest correlate of DBP.

**Table 3:** Pearson's correlation of SBP and DBP with some clinical variables

Variables	Systolic blood pressure		Diastolic blood pressure	
	*r*	*P* value	*r*	*P* value
Age	0.33	< 0.001	0.17	< 0.001
WC	0.23	< 0.001	0.22	< 0.001
Weight	0.14	< 0.001	0.21	< 0.001
BMI	0.15	< 0.001	0.18	< 0.001

### Predictors of Hypertension

The significant factors from the bivariate analyses were entered into a multivariate binary logistic regression model with hypertension status as dependent variable (Table 4). There was no multicollinearity among the variables included in the model. The predictors of hypertension among the respondents include increasing age, high waist circumference and high BMI. Compared to those aged 20–39 years, the participants aged 60 and above were more than 10 times likely to have hypertension. In the same vein, respondents with high waist circumference and those with BMI greater than 25kg/m^2^ had higher odds of having hypertension.

**Table 4:** Multivariate binary logistic regression table showing the predictors of hypertension among the respondents

	Beta	P value	Odds ratio	95% Confidence Interval
**Age group**				
20–39 (reference)			1	
40–59	1.31	< 0.001	3.70	2.55 – 5.36
60 above	2.34	< 0.001	10.36	6.85 – 15.66
**Marital status**				
Single (reference)			1	
Married	-0.16	0.63	0.86	0.45 – 1.62
Widowed	-0.13	0.74	0.88	0.40 – 1.92
**Waist circumference**				
Normal (reference)			1	
High	0.62	0.01	1.86	1.18 – 2.95
**BMI**				
< 25 kg/m^2^ (reference)			1	
> 25 kg/m^2^	0.50	0.03	1.65	1.04 – 2.60

**Note**: Gender was not included in multivariate model. It was not significant in bivariate analysis.

## Discussion

The overall prevalence of hypertension in our study was 45.6% which is quite high but closely agrees with reports of 47.2%^14^, 47%^15^, and 46.4%^5^ from similar studies in rural communities across different parts of the country. It is however much higher than what was documented by some other authors in similar rural settings^6^,^16^,^17^.

In a systematic review of the prevalence of hypertension in Nigeria, Akinlua et al.^8^ noted the existence of wide variations in reported values across different studies. Some of these differences may be accounted for by methodological variations especially with regards to the age composition of studied population. For example, one of the studies^6^ which reported a lower prevalence compared to ours, included participants as young as 15 years and the mean age of their subjects was 32.3 years which is much lower than that of this study.

At the bivariate level of analysis, this study found that, in addition to age, the presence of hypertension was significantly associated with marital status, high BMI and central obesity. Other studies have reported similar findings^16^,^18^–^21^. It is a well-known fact that hypertension increases with age and this has been documented in virtually all previous studies^18^–^21^. The prevalence of hypertension increased from about 12% in age group 20–29 years to 73% in those aged 60–69 years, before dropping slightly to 67.2% in those above 70 years. This is similar to the pattern documented by Adediran et al.^20^.

Furthermore, age was found to be the strongest correlate of SBP in this study. With increasing age, there is a decline in arterial compliance with resultant increase in peripheral resistance. This tends to affect the SBP more substantially^5^. This pattern is in consonance with what has been described in previous reports^5^,^20^. This is further corroborated by the fact ISH was more prevalent among those who were 60 years and above. Isolated systolic hypertension in the elderly has been shown to be associated with substantial morbidity and mortality^22^. The presence of hypertension was also found to be significantly associated with marital status from bivariate statistical analysis. The prevalence was highest in the widowed subjects. This is most likely due to the fact that those in this category were likely to also be older in age, which probably explains why this bivariate statistical significance was not sustained after controlling for age and other significant factors in a multivariate analysis.

The multivariate binary logistic regression analysis revealed, in line with some earlier reports^15^,^18^ that age, waist circumference and body mass index were independent predictors of hypertension in the study population.

It must however be noted that the cross-sectional nature of this study limits the establishment of causal relationship between the hypertension and the identified factors. Furthermore, some other potentially confounding variables such as socio-economic status, lifestyle factors, presence of comorbidities e.g. diabetes, were not included in the model. Another limitation is the fact that hypertension was defined on the basis of measurement of blood pressure at a single contact which may affect the prevalence^23^. The finding of blood pressure consistent with stage 1 or mild hypertension at an initial contact require confirmation at another follow-up contact within one month^23^. The difficulty in determining the ages of some respondents was also a limitation- Some, especially those who were elderly, did not have records for an accurate determination of their ages, hence estimations were done using historical events.

## Conclusion

There was a high prevalence of hypertension in the study population and the predictive factors were age, BMI and waist circumference. There is the need for concerted efforts by health policy makers and all stakeholders towards putting in place effective primary and secondary preventive strategies. Modifiable risk factors such as obesity need to be reduced through appropriate public health promotion measures. The rural areas require an increased share of such attention than they currently receive.
